# Supplementary material for: Mitochondrial Fission Process 1 controls inner membrane integrity and protects against heart failure
Source: Nat Commun. 2022 Nov 4;13:6634. doi: 10.1038/s41467-022-34316-3 (PMC9636241; doi:10.1038/s41467-022-34316-3)
Supplement: Supplementary file 1 — Supplementary Information [file 41467_2022_34316_MOESM1_ESM.pdf]

Supplementary Information

Mitochondrial Fission Process 1 controls inner membrane integrity and protects against heart failure.

Erminia DONNARUMMA, Michael KOHLHAAS, Elodie VIMONT, Etienne KORNOBIS, Thibault CHAZE, Quentin GIAI GIANETTO, Mariette MATONDO, Maryse MOYA-NILGES, Christoph MAACK, Timothy WAI

Supplementary information

- Supplementary Figures 1-6
- Supplementary Table 1
- Supplementary Data 1 Cardiac Proteome - WT vs cMKO
- Supplementary Data 2 RNAseq - WT vs cMKO
- Supplementary Data 3 Cardiac CoIP WT vs FLAG-MTFP1
- Supplementary Data 4 Reagents

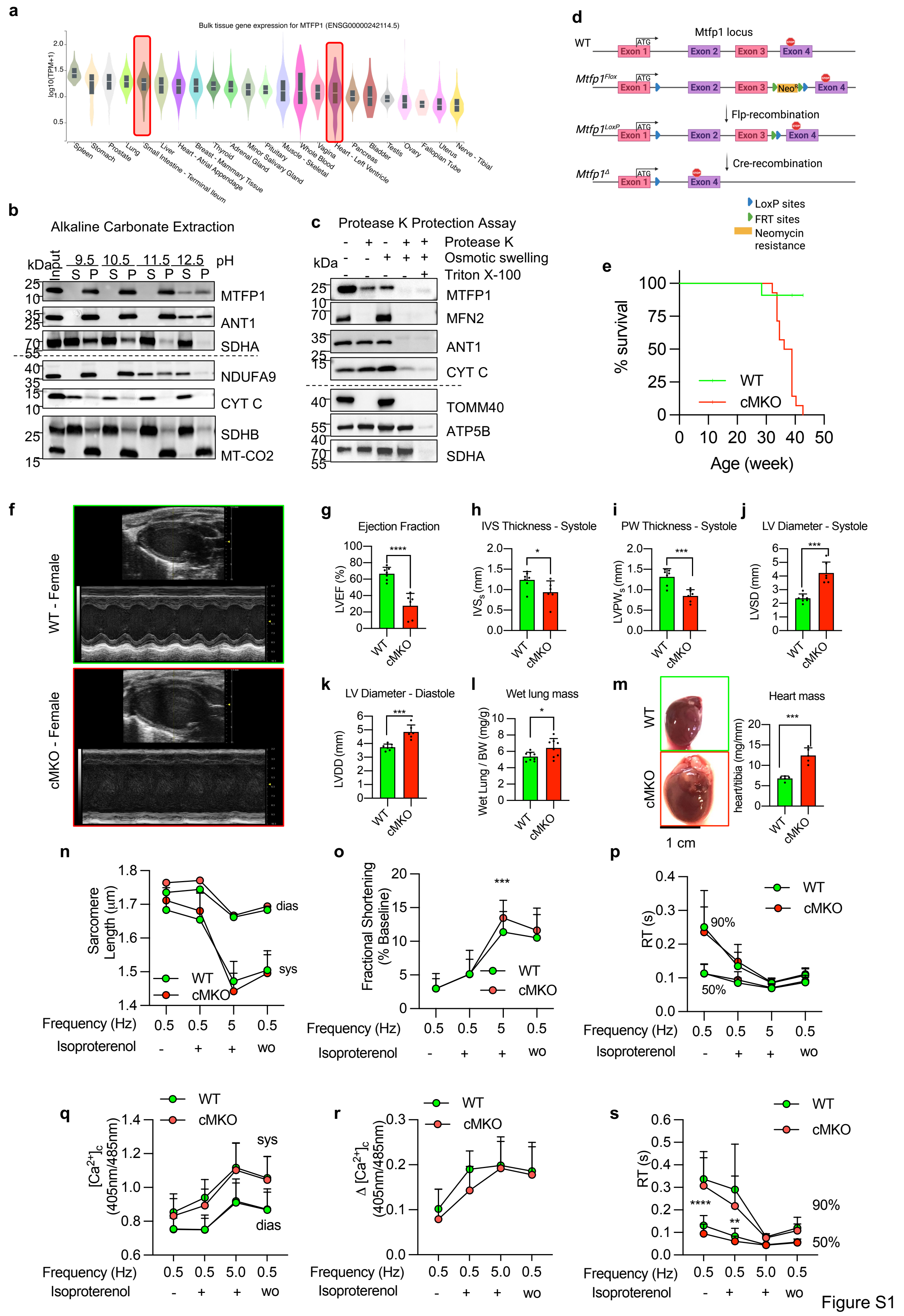

***Figure S1*. *Mtfp1* deletion in cardiomyocytes causes dilated cardiomyopathy and middle-aged death in mice**

a) Genotype tissue expression plot of MTFP1 in human tissues. **b)** Immunoblot of soluble (S) and insoluble (pellet, P) fractions of wild type (WT) cardiac mitochondria extracted with Na_2CO_3 at the

indicated pH. Experiment was performed once, antibodies as indicated. **c)** Determination of sub-mitochondrial localization of MTFP1 in cardiac mitochondria by protease K protection assay performed once, antibodies as indicated. **d)** Targeting strategy for conditional inactivation of mouse *Mtfp1*.**e)** Kaplan-Meier survival curve of WT (n=11) and cMKO (n=14) female mice. Median lifespan of cMKO mice is 37.5 weeks. **f)** M-Mode echocardiography of WT (top) and cMKO (bottom) female mice at 34 weeks. **g)** Left ventricular ejection fraction (% LVEF), **h)** Systolic interventricular septum thickness (IVSs, mm), **i)** Systolic left ventricular posterior wall thickness (LVPWs, mm), **j)** Left ventricle end systolic diameter (LVSD, mm), **k)** Left ventricle end diastolic diameter (LVDD, mm) of WT (n=7) and cMKO (n=6) female mice at 34 weeks. Data represent mean ± SD. 2-tailed unpaired Student's t-test, *p<0.05, ***p<0.001, ****p<0.0001. **l)** Wet Lung/Body Weight (BW) of WT (n=8) and cMKO (n= 8) at 34 weeks. Data represent mean ± SD; 2-tailed unpaired Student's t-test, *p<0.05.**m)** Representative images (left) of WT (top, n=5) and cMKO (bottom, n=5) hearts isolated from female mice at 34 weeks; (right) heart mass to tibia length (mg/mm). Data represent mean ± SD, 2-tailed unpaired Student's t-test, ***p<0.001. **n-s)** Isolated primary adult cardiomyocytes from WT (n=51) and cMKO (n=52) mice at 8 weeks were i) field-stimulated at 0.5 Hz, ii) exposed to isoproterenol and iii) stimulated at frequency of 5 Hz, iv) and then stepped back to 0.5 Hz washing out (wo) isoproterenol. **n)** Diastolic and systolic sarcomere length (μm) of WT and cMKO; **o**) fractional sarcomere shortening; **p)** Time to 50% or 90% decay of sarcomere shortening in WT or cMKO myocytes, ***p<0.001. **q-s)** Isolated primary adult cardiomyocytes from WT (n=29) and cMKO (n=52) mice at 8 weeks were i) field-stimulated at 0.5 Hz, ii) exposed to isoproterenol and iii) stimulated at frequency of 5 Hz, iv) and then stepped back to 0.5 Hz washing out (wo) isoproterenol. Intracellular Ca^{2+} concentrations ($[Ca^{2+}]_c$) were assessed by loading cells with Indo-1 AM and recording calcium transients at 405 nm/485 nm. **q)** $[Ca^{2+}]_c$ at diastole or systole; **r)** amplitude of $[Ca^{2+}]_c$ transients; **s**) Time to 50% or 90% decay (RT) of $[Ca^{2+}]_c$ in WT and cMKO myocytes, **p<0.01, ****p<0.0001. Data represent mean ± SD.

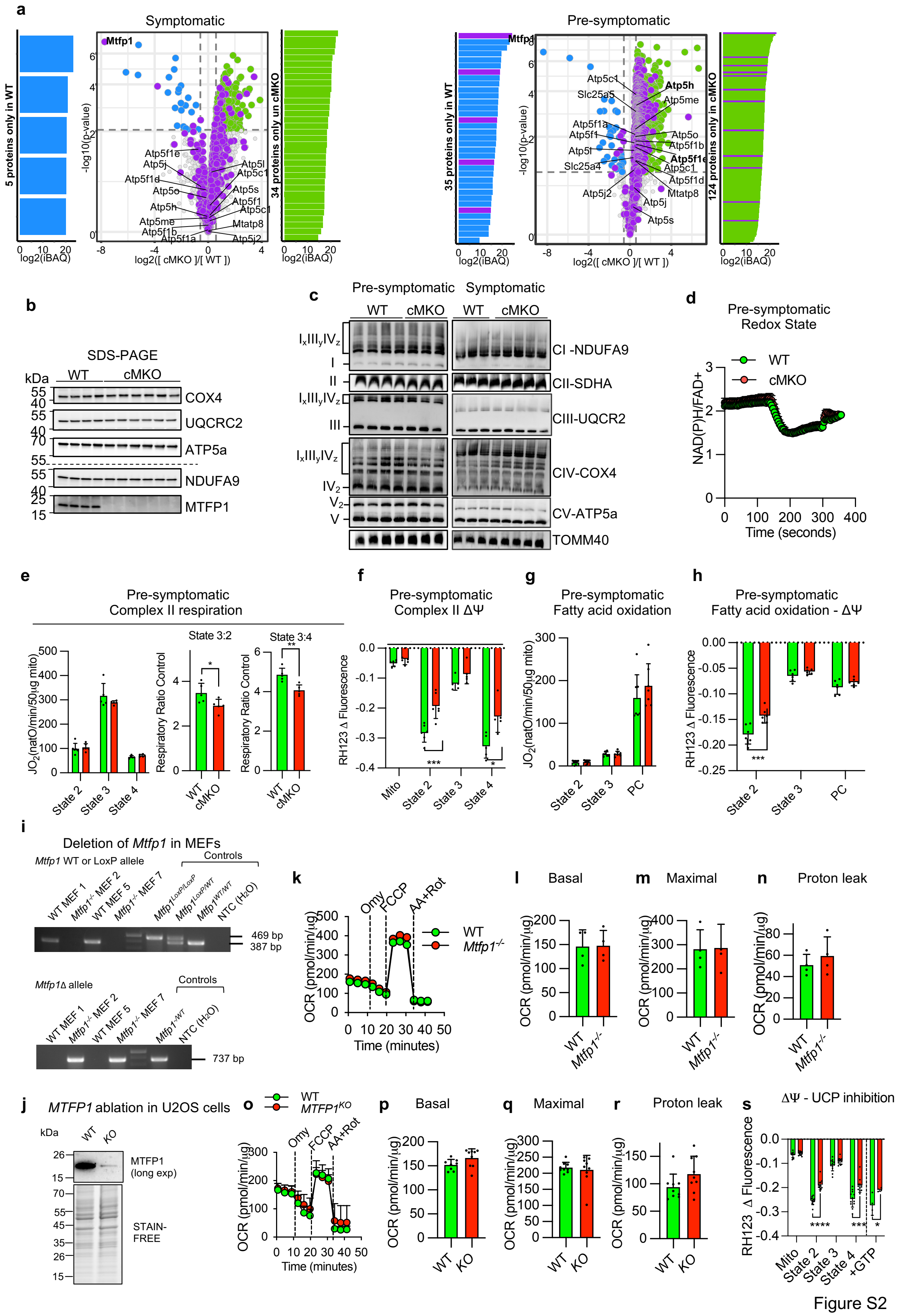

Figure S2

Figure S2. Mtfp1 is required for bioenergetic efficiency in cardiac mitochondria

a) Volcano plot of the cardiac proteome in WT and cMKO mice at symptomatic (18 weeks, left) and pre-symptomatic (8-10 weeks, right) listed in Supplementary Data 1. Mitochondrial proteins (purple) and non-mito proteins significantly up-regulated (green) and down-regulated (blue).
b) Immunoblots from male WT (n=4) and cMKO (n=6) hearts at 18 weeks analyzed with the indicated antibodies. **c)** BN-PAGE immunoblot analysis of cardiac OXPHOS complexes isolated from male

WT and cMKO mice at 8-10 weeks (pre-symptomatic, WT n=4, cMKO n=3) and at 30 weeks (symptomatic, WT n=3, cMKO n=5, right) using the indicated antibodies. **d)** Ratio of NAD(P)H/FAD of the redox states of NAD(P)H/NAD(P)$^+$ and $FADH_2$/FAD assessed in field-stimulated WT (n=51) and cMKO (n=51) cardiomyocytes isolated from 8-10 weeks old female mice (n=3). Data represent mean ± SEM. **e)** Oxygen consumption rates (left; JO_2) of cardiac mitochondria from WT (n=5) and cMKO (n=5) female mice between 8-10 weeks measured in presence of succinate and rotenone (state 2) followed by the addition of ADP (state 3) and Oligomycin (Omy- state 4). Data represent mean ± SD. Respiratory control ratios (RCR) of state 3:2 (middle; ADP/Succinate (SUCC)) and state 3:4 (right; ADP/Omy) under complex-II driven respiration. Data represent mean ± SD; multiple t-test, *p<0.05, **p<0.01. **f)** Mitochondrial membrane potential ($\Delta\Psi$) measured by quenching of Rhodamine 123 (RH123) fluorescence in cardiac mitochondria from WT (n=8) and cMKO (n=8) female mice between 8-10 weeks. $\Delta\Psi$ was measured in presence of rotenone and succinate (state 2) followed by the addition of ADP (state 3) and Oligomycin (state 4). Data represent mean ± SD; Multiple t-test, *p<0.05, ***p<0.001. **g)** Oxygen consumption rates (JO_2) of cardiac mitochondria isolated from WT (n=6) and cMKO (n=6) female mice between 8-10 weeks measured in presence of malate (state 2) followed by the addition of ADP (state 3) and palmitoyl-carnitine (PC). Data represent mean ± SD. **h)** Mitochondrial membrane potential ($\Delta\Psi$) measured by quenching of Rhodamine 123 (RH123) fluorescence in cardiac mitochondria isolated from WT (n=6) and cMKO (n=6) female mice between 8-10 weeks. $\Delta\Psi$ was measured in presence of malate (state 2) followed by the addition of ADP (state 3) and palmitoyl-carnitine (PC). Data represent mean ± SD; Multiple t-test, ***p<0.001. **i)** PCR genotyping of *Mtfp1* alleles in mice. Deletion of exons 2 and 3 was obtained by crossing *Mtfp1*$^{LoxP/LoxP}$ mice with *CMV-Cre* recombinase mice to generate heterozygous knockout *Mtfp1*$^{+/-}$ offspring. The wild type (387 bp) and the deleted (737 bp) alleles are shown for *Mtfp1*$^{+/+}$ and *Mtfp1*$^{-/-}$ cells. **j)** Equal amounts of protein extracted from WT and *MTFP1*^{KO} U2OS cells separated by SDS–PAGE and immunoblotted with antibodies against MTFP1 and normalized against Stain Free. Experiment was performed 4 times with a similar result. **k-n**) Mitochondrial respiration measured in wild type (WT) and *Mtfp1*$^{-/-}$ MEF cells using the Seahorse Flux Analyzer. **l)** Oxygen consumption rate (OCR) normalized to protein concentration under **l)** Basal **m)** Maximal (FCCP) and **n)** Proton leak. Data are means ± SD of n=4 independent experiments measured on different days. Each point represents the mean of 7-12 technical OCR measurements replicates of each independent experiment. **p-r)** Mitochondrial respiration measured in wild type (WT) and *MTFP1*^{KO} human U2OS osteosarcoma cells using the Seahorse Flux Analyzer. **o)** Oxygen consumption rate (OCR) normalized to protein concentration under **p)** Basal **q)** Maximal (FCCP) and **r)** Proton leak. Data are means ± SD of 9 individually plated wells. **s)** Mitochondrial membrane potential ($\Delta\Psi$) measured by quenching of Rhodamine 123 (RH123) fluorescence in cardiac mitochondria of WT and cMKO female mice between 8-10 week of age. $\Delta\Psi$ was measured in presence of pyruvate, malate, and glutamate (state 2) followed by the addition of ADP (state 3), oligomycin (state 4) and GTP [WT (n=3), cMKO (n=3)]. State 2, 3, and 4 data are represented in Figure 2E as a reference. Data are mean ± SD; Multiple t-test, *p<0.05, ***p<0.001, ****p<0.0001.

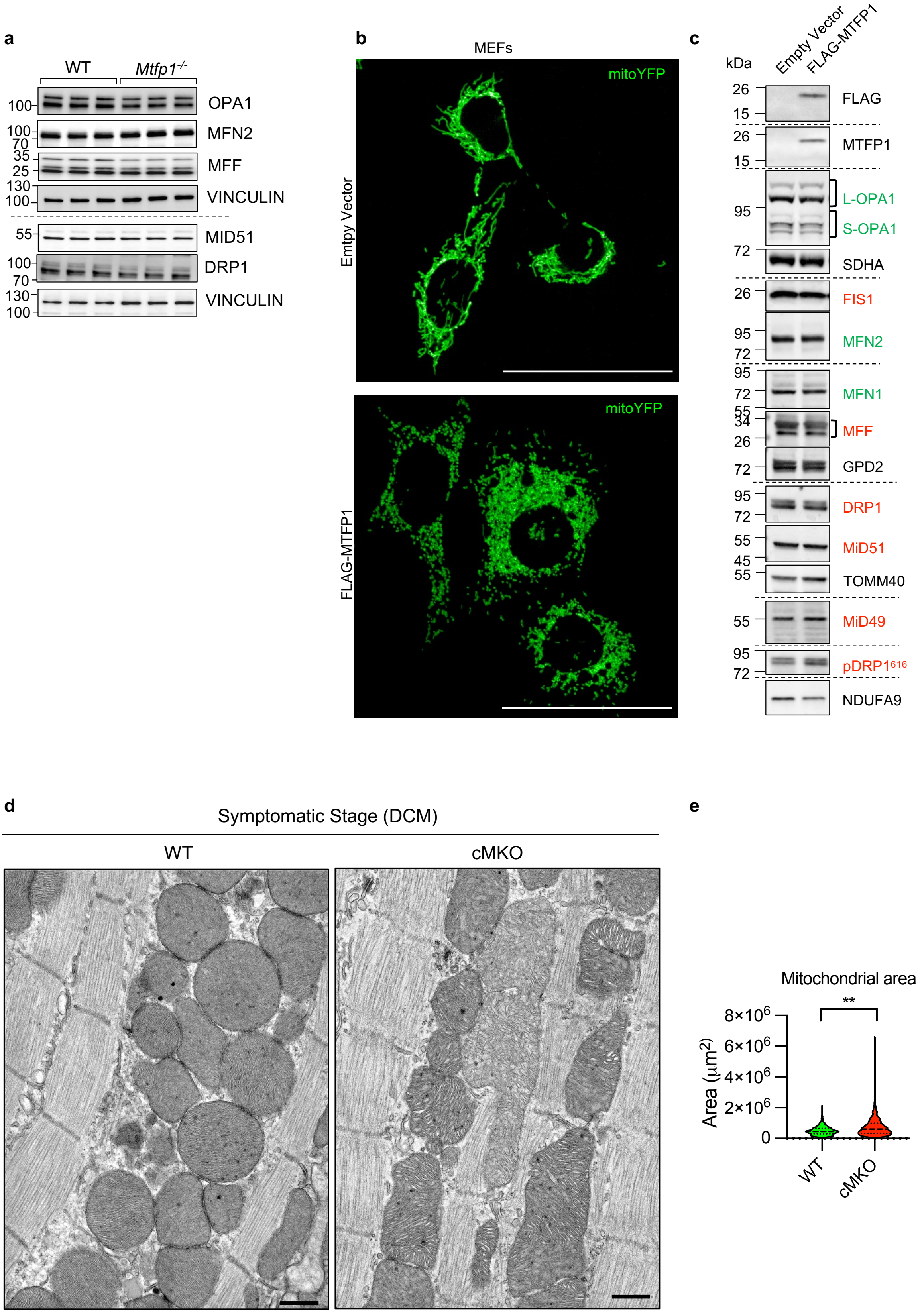

Figure S3

Figure S3. MTFP1 is dispensable for mitochondrial fission.

a) Immunoblot of mitochondrial fission and fusion proteins in WT and *Mtfp1*$^{-/-}$ MEFs. Equal amounts of protein extracted from WT and *Mtfp1*$^{-/-}$ MEFs in three independent experiments were separated by SDS–PAGE and immunoblotted with the indicated antibodies (horizontal line denotes different membranes). VINCULIN was used as loading control. **b)** Representative confocal images of fragmented mitochondria morphology in MEFs stably expressing FLAG-MTFP1 (mitoYFP, green) or

the Empty Vector control. Scale bar=100 µm. Experiment was performed 6 times with a similar result. **c)** Representative immunoblot of mitochondrial fission (red) and fusion (green) proteins in MEFs stably overexpressing FLAG-MTFP1 or the Empty Vector control. Equal amounts of protein were separated by SDS–PAGE and immunoblotted with the indicated antibodies (horizontal line denotes different membranes). Experiment was performed 3 times with a similar result**. d)** Representative transmission electron micrographs of cardiac posterior walls of WT (left, n=2) and cMKO (right, n=3) mice at 18 weeks. Scale bar: 500 nm. **e)** Violin plot of mitochondrial surface area (µm^2) quantified within cardiac posterior wall measured in D (WT mitochondria n=837; cMKO mitochondria n=1730). Dotted line represents quartiles and dashed line represents median; **p<0.01 Mann-Whitney test.

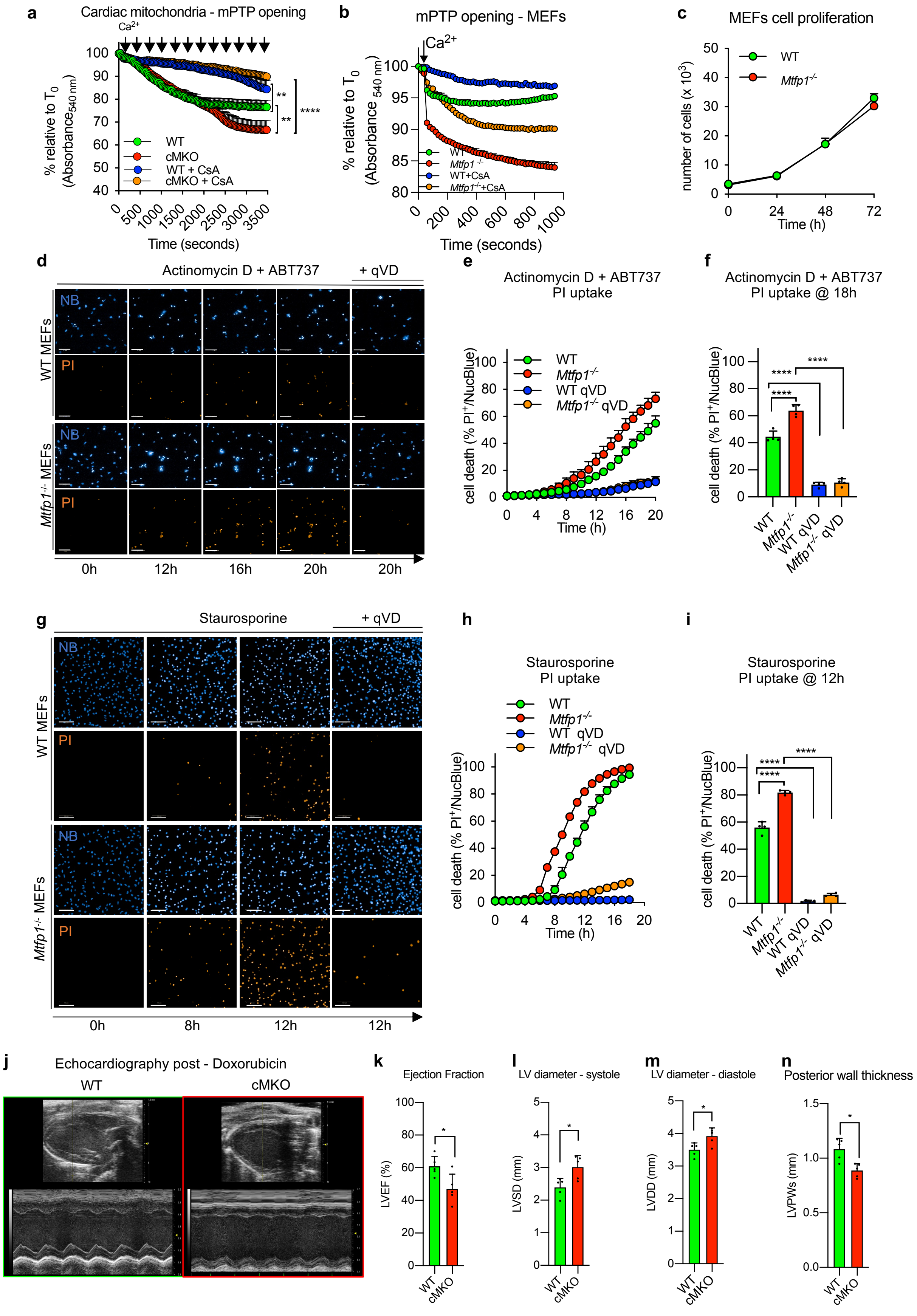

Figure S4

Figure S4. MTFP1 protects against mitochondrial PTP opening and cell death

a) Mitochondrial swelling assay of cardiac mitochondria from hearts of WT (n=3) and cMKO (n=3) female mice at 8-10 weeks and **b**) mitochondria purified from WT and *Mtfp1*$^{-/-}$ MEFs. Relative absorbance at 540 nm was measured every 20 s a) or 30 s b) before and after addition of a single or multiple pulses of $CaCl_2$ (arrowhead) in presence or absence of Cyclosporin A (CsA). Data are

means ± SD of 2 technical replicates for each condition. One-way ANOVA of maximal absorbance 540 nm (%relative to T0) change, **p<0.01, **** p<0.0001.

c) Cell proliferation curves of WT and *Mtfp1*$^{-/-}$ MEFs in glucose (4.5 g/L) labeled with NucBlue (NB). Data represent mean ± SD of n=16 individually plated wells. **d)**Representative images of WT and *Mtfp1*$^{-/-}$ MEFs subjected to actinomycin D (ActD) and ABT-737 with or without q-VD-OPh hydrate (qVD). Cell death was monitored by using nuclear Propidium Iodide uptake (PI, orange) every hour (h) for 20h. Scale bar = 100 µm. **e)** Cell death defined by the number of PI^+ positive cells (orange) over total number cells nuclear stained with NucBlue (NB, blue) and expressed as % PI^+/NucBlue. Data are means ± SD of n=4 independent experiments. **f)** one-way ANOVA of e) at 18h, ****p<0.0001. **g)** Representative images of WT and *Mtfp1*$^{-/-}$ MEFs subjected to staurosporine (STS) with or without q-VD-OPh hydrate (qVD). Cell death was monitored by measuring Propidium Iodide uptake (PI) and imaging cells every hour (h) for 18 h. Scale bar = 100 µm. **h)** Cell death defined by the number of of PI^+ positive cells (orange) over total number cells nuclear stained with NucBlue (NB, blue) and expressed as % PI^+/NucBlue. Data are means ± SD of n=4 independent experiments. **i)** one-way ANOVA of h) at 12h, ****p<0.0001. **j)** Representative M-Mode echocardiographic images of left ventricles of WT (left) and cMKO (right) mice treated with doxorubicin (Doxo, 20 mg/kg) at 10 weeks. **k)** Left ventricular ejection fraction (% LVEF), **l)** Systolic left ventricular diameter (LVSD, mm), **m)** Diastolic left ventricular diameter (LVDD, mm) **n)** Systolic left ventricular posterior wall thickness (LVPWs, mm) of WT (n=5) and cMKO (n=5) after 2 weeks of Doxo administration. Data represent mean ± SD. 2-tailed unpaired Student's t test, *p<0.05.

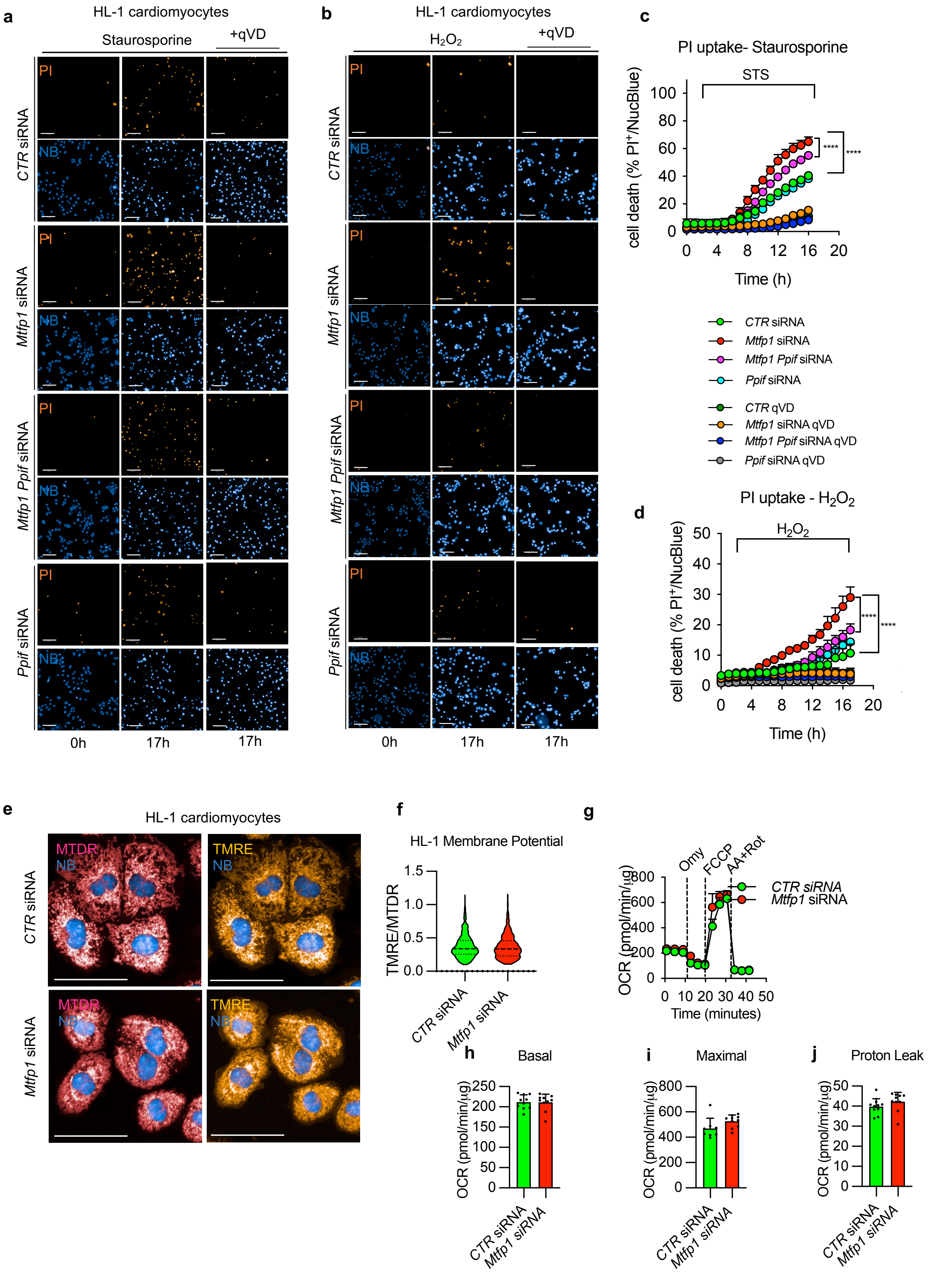

Figure S5

Figure S5. MTFP1 protects against mitochondrial PTP mediated cell death in HL-1 cells

a) Representative images of HL-1 cardiomyocytes treated with the indicated siRNA subjected to staurosporine (STS) treatment with or without the pan-caspase inhibitor q-VD-OPh hydrate (qVD). Cell death was monitored by measuring Propidium Iodide uptake (PI) and imaging cells every hour (h) for 17 h on n=2 (CTR) and n=4 (siRNA) independent replicates. Scale bar = 100 μm. **b)** Representative images of HL-1 cardiomyocytes treated with the indicated siRNA subjected to H_2O_2

treatment with or without q-VD-OPh hydrate (qVD). Cell death was monitored by measuring Propidium Iodide uptake (PI) and imaging cells every hour (h) for 17 h on n=2 (CTR) and n=4 (siRNA) independent replicates. Scale bar = 100 μm. **c)** Kinetics of PI uptake in a) was determined by counting the number of PI^+ positive cells (orange) over total number cells nuclear stained with NucBlue (NB, blue) and expressed as % PI^+/NucBlue. Data are means ± SD of n=2 (CTR) and n=4 (siRNA) independent replicates. **d)** Kinetics of PI uptake in b) was determined by counting the number of PI^+ positive cells (orange) over total number cells nuclear stained with NucBlue (NB, blue) and expressed as % PI^+/NucBlue. Data are means ± SD of n=4 independent experiments. **e)** Representative images of HL-1 cardiomyocytes treated with indicated siRNAs were labeled with tetramethylrhodamine ethyl ester (TMRE, orange), MitoTracker Deep Red (MTDR, red) and NucBlue (NB, blue). Scale bar=100 μm. **f)** Violin plot of mitochondrial membrane potential of HL-1 cardiomyocytes treated with the indicated siRNA. TMRE/MTDR intensity per cell (CTR siRNA n=1368, Mtfp1 siRNA n=589) calculated from e). Dotted line represents quartiles and dashed line represents median. **g-j)** Mitochondrial respiration measured in HL-1 cardiomyocytes after 72h treatment with the indicated siRNA using the Seahorse Flux Analyzer. **g)** Oxygen consumption rate (OCR) normalized to protein concentration under **h)** Basal **i)** Maximal (FCCP) and **j)** Proton leak. Data are means ± SD of n=10 independent replicates. Each point represents the technical replicates of the OCR measurements.

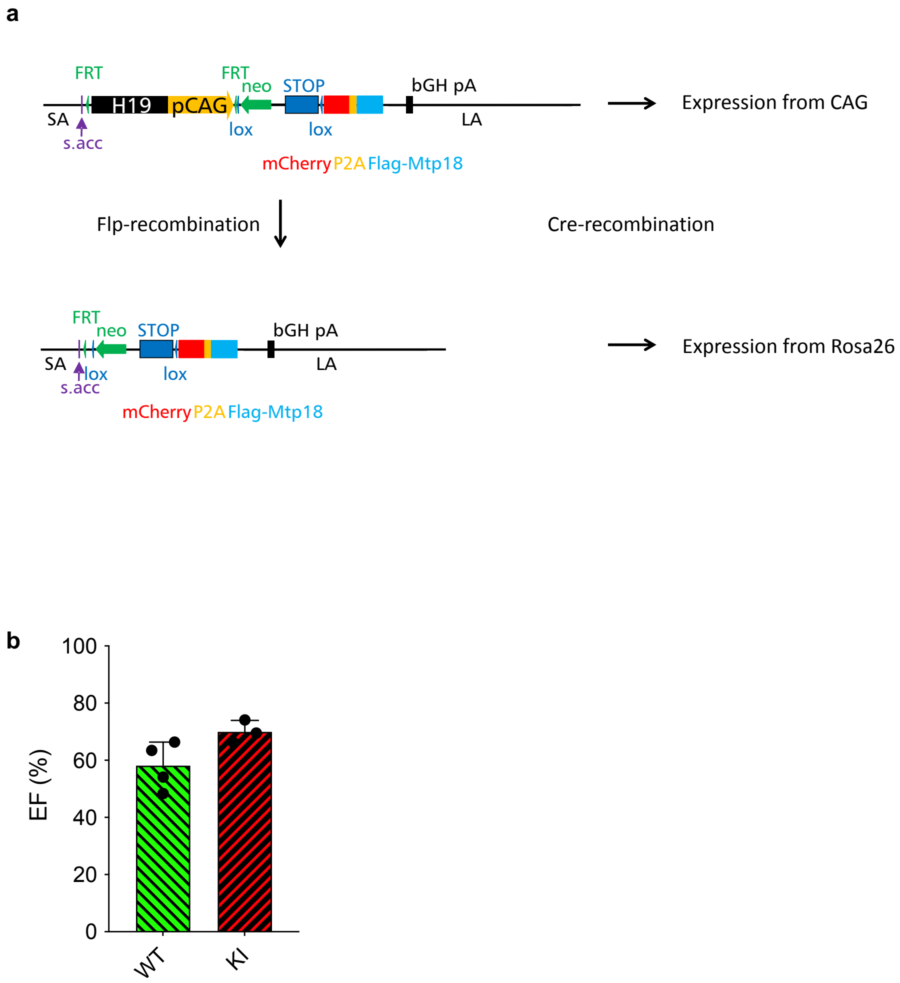

Figure S6. Generation of FLAG-MTFP1 mouse model to define the cardiac interactome

a) For inducible overexpression of MTFP1 (mCherry-P2A-Flag-Mtp18), two mouse models were generated from one construct. The first (upper pane) allowing inducible expression from the CAG-promoter, the second (lower panel) from the mouse Rosa26 promoter. To do so, the construct in the upper panel is generated and recombined into the mouse Rosa26 locus. The genuine Rosa26 promoter is further upstream, and its transcript is spliced into the splice acceptor of exon 2 (“s.acc.”, purple). In the native state, transcription is aborted at the insulator site (H19, black), and instead started at the CAG promoter (pCAG, yellow arrow). The loxP-flanked (lox, blue arrow heads) neo–STOP cassette for conditional activation has a stop site that is a large region of SV40 intron plus late polyA (neo, green arrow; STOP, blue box). The cDNA is inserted downstream of the neo/stop cassette. Both, Rosa26-driven and CAG-driven expression, is polyadenylated at a bovine growth hormone polyA site (bGH pA, small black box). For exchange of the promoters, the CAG-cassette is flanked by FRT-sites. This cassette will be excised by Flp-mediated deletion in vivo, leaving mCherry-P2A-Flag-Mtp18 expression under control of the Rosa26 promoter. Upon Cre mediated deletion, the mCherry-P2A-Flag-Mtp18 cDNA is expressed from the CAG promoter (upper panel) or the intrinsic Rosa26 promoter (lower panel). **b)** Quantification of left ventricular ejection fraction (% LVEF) of WT (n=4) and KI (n=3) male mice at 20 weeks. Data represent mean ± SD.

Pre-symptomatic (8-10wk)	WT	cMKO	p value
Complex I - NDUFA9	1.64±0.138	1.58±0.164	0.628
Complex II - SDHA	2.25±0.947	1.70±0.177	0.378
Complex III - UQCR2	0.711±0.0892	0.528±0.141	0.0885
Complex IV - COX4	15.41±3.31	12.98±1.096	0.285
Complex V - ATP5A	1.02±0.155	0.812±0.0475	0.0834
Complex V2 - ATP5A	0.622±0.122	0.524±0.0945	0.304

Symptomatic (30 wk)	WT	cMKO	p value
Complex I - NDUFA9	3.85±0.457	3.12±0.710	0.167
Complex II - SDHA	3.51±0.395	2.96±0.394	0.106
Complex III - UQCR2	2.08±0.0904	1.458±0.377	**0.0337**
Complex IV - COX4	13.0±0.145	11.0±2.79	0.291
Complex V - ATP5A	2.46±0.344	1.79±0.322	**0.0328**
Complex V2 - ATP5A	0.891±0.106	0.583±0.102	0.0743

Supplementary Table 1. Quantification of Supplementary Figure S2C
