# Supplementary material for: Mitochondrial Fission Process 1 controls inner membrane integrity and protects against heart failure
Source: Nat Commun. 2022 Nov 4;13:6634. doi: 10.1038/s41467-022-34316-3 (PMC9636241; doi:10.1038/s41467-022-34316-3)
Supplement: Supplementary file 3 — Description of Additional Supplementary Files [file 41467_2022_34316_MOESM3_ESM.pdf]

Description of additional supplementary files

Supplementary Data 1: Cardiac Proteome - WT vs cMKO

Supplementary Data 2: RNAseq - WT vs cMKO

Supplementary Data 3: Cardiac CoIP WT vs FLAG-MTFP1

Supplementary Data 4: Reagents
