# Supplementary material for: Mitochondrial Fission Process 1 controls inner membrane integrity and protects against heart failure
Source: Nat Commun. 2022 Nov 4;13:6634. doi: 10.1038/s41467-022-34316-3 (PMC9636241; doi:10.1038/s41467-022-34316-3)
Supplement: Supplementary file 8 — Reporting Summary [file 41467_2022_34316_MOESM8_ESM.pdf]

nature portfolio

Corresponding author(s): TIMOTHY WAI

Last updated by author(s): Oct 6, 2022

Reporting Summary

Nature Portfolio wishes to improve the reproducibility of the work that we publish. This form provides structure for consistency and transparency in reporting. For further information on Nature Portfolio policies, see our Editorial Policies and the Editorial Policy Checklist.

Statistics

For all statistical analyses, confirm that the following items are present in the figure legend, table legend, main text, or Methods section.

n/a	Confirmed	
☐	☒	The exact sample size (*n*) for each experimental group/condition, given as a discrete number and unit of measurement
☐	☒	A statement on whether measurements were taken from distinct samples or whether the same sample was measured repeatedly
☐	☒	The statistical test(s) used AND whether they are one- or two-sided<br>*Only common tests should be described solely by name; describe more complex techniques in the Methods section.*
☐	☒	A description of all covariates tested
☐	☒	A description of any assumptions or corrections, such as tests of normality and adjustment for multiple comparisons
☐	☒	A full description of the statistical parameters including central tendency (e.g. means) or other basic estimates (e.g. regression coefficient) AND variation (e.g. standard deviation) or associated estimates of uncertainty (e.g. confidence intervals)
☐	☒	For null hypothesis testing, the test statistic (e.g. *F*, *t*, *r*) with confidence intervals, effect sizes, degrees of freedom and *P* value noted<br>*Give P values as exact values whenever suitable.*
☒	☐	For Bayesian analysis, information on the choice of priors and Markov chain Monte Carlo settings
☒	☐	For hierarchical and complex designs, identification of the appropriate level for tests and full reporting of outcomes
☒	☐	Estimates of effect sizes (e.g. Cohen's *d*, Pearson's *r*), indicating how they were calculated

Our web collection on statistics for biologists contains articles on many of the points above.

Software and code

Policy information about availability of computer code

Data collection	Commercially available software was used for the collection of cardiac ultrasound (Vevo 3100, VisualSonics), fluorescence microscopy (Harmony 4.9, Perkin Elmer), fluorespirometry (O2k-FlouRespirometer, Oroboros), immunoblotting (Bio-Rad Chemidoc), and electron microscopy (Serial EM), and immunohistochemistry (OlyVIA, Olympus)
Data analysis	Commercially available software was used for the analysis of cardiac ultrasound (Vevo Lab,VisualSonics), fluorespirometry (Data Lab Oroboros), immunoblotting (Imagelab, Bio-Rad), fluorescence microscopy (Harmony 4.9, Perkin Elmer), and electron microscopy data (3dmod). Statistical analyses were performed using Graph Pad Prism v9.

For manuscripts utilizing custom algorithms or software that are central to the research but not yet described in published literature, software must be made available to editors and reviewers. We strongly encourage code deposition in a community repository (e.g. GitHub). See the Nature Portfolio guidelines for submitting code & software for further information.

Data

Policy information about availability of data

All manuscripts must include a data availability statement. This statement should provide the following information, where applicable:

- Accession codes, unique identifiers, or web links for publicly available datasets
- A description of any restrictions on data availability
- For clinical datasets or third party data, please ensure that the statement adheres to our policy

Source Data are provided with this paper. The datasets generated during the current study are available in the European Nucleotide Archive (NEO) repository and

Proteomics Identification Database (PRIDE). Accession numbers and the web links are as follows. Bulk RNAseq: (ENA: Project: PRJEB47968), Cardiac proteome of mice at pre-symptomatic (PXD028516??COMPLETE) and symptomatic (???PXD028516) ages, and cardiac interactome data (PXD028529).The total datasets of fluorescence microscopy images generated and analyzed during the current study are not publicly available due to the incompatibility of exporting comprehensible file names linked to cell, treatment, and time identifiers from the Harmony 4.9, Perkin Elmer software but are available from the corresponding author on reasonable request.

Human research participants

Policy information about studies involving human research participants and Sex and Gender in Research.

Reporting on sex and gender	*Use the terms sex (biological attribute) and gender (shaped by social and cultural circumstances) carefully in order to avoid confusing both terms. Indicate if findings apply to only one sex or gender; describe whether sex and gender were considered in study design whether sex and/or gender was determined based on self-reporting or assigned and methods used. Provide in the source data disaggregated sex and gender data where this information has been collected, and consent has been obtained for sharing of individual-level data; provide overall numbers in this Reporting Summary. Please state if this information has not been collected. Report sex- and gender-based analyses where performed, justify reasons for lack of sex- and gender-based analysis.*
Population characteristics	*Describe the covariate-relevant population characteristics of the human research participants (e.g. age, genotypic information, past and current diagnosis and treatment categories). If you filled out the behavioural & social sciences study design questions and have nothing to add here, write "See above."*
Recruitment	*Describe how participants were recruited. Outline any potential self-selection bias or other biases that may be present and how these are likely to impact results.*
Ethics oversight	*Identify the organization(s) that approved the study protocol.*

Note that full information on the approval of the study protocol must also be provided in the manuscript.

Field-specific reporting

Please select the one below that is the best fit for your research. If you are not sure, read the appropriate sections before making your selection.

☒ Life sciences ☐ Behavioural & social sciences ☐ Ecological, evolutionary & environmental sciences

For a reference copy of the document with all sections, see nature.com/documents/nr-reporting-summary-flat.pdf

Life sciences study design

All studies must disclose on these points even when the disclosure is negative.

Sample size	Sample sizes were determined to be adequate based on the literature describing similar experiments (Wai et al. Science 2015), and magnitude and consistency of measurable differences between groups, power studies (performed in collaboration with Biostatisticians at the Institut Pasteur), and space available in an animal containment facilities used for breeding (BIME) and experimentation (Monod).
Data exclusions	No data points were excluded in this study except for 2 out of 10 replicate wells at indicated time points in Figure S5g (see source data).
Replication	Animal experiments were repeated at least three times on independent biological samples. Biological assays (ELISA) were performed in 2 technical replicates on at least n=3 independent samples. Ex vivo and in vitro experiments (cell death/growth assays, seahorse,biochemical assays) were conducted at least on two or three biologically independent samples with reproducible data. All attempts at replication were successful and the replicates have been now reported in the figure legends.
Randomization	For animal studies, control and mutant mice were randomly allocated to cages and separated by gender at the time of weaning. Physiological measurements were made at random, cage-by-cage. For pair-wise experimental measurements performed in vitro (e.g. Oroboros, cardiomyocyte isolation, electro-physiology), mutant and wild type samples were measured in parallel.For experiments other than animal studies, samples were allocated randomly between genotypes where possible. Allocation was not random when simultaneous, pairwise measurements (e.g. machine with only two chambers) was obliged. In this case, randomly selected wild type control and randomly selected mutant samples were measured in parallel in individual chambers.
Blinding	Blinding was performed whenever possible (namely for all animal experiments), which excluded experiments in which experimental protocols only allowed for parallel, pair-wise measurements (see above), in which case random wild type and mutant samples were measured from cells or animals that had previously been genotyped. Cell and tissue image acquisition and analyses were performed automatically and the experimentalist was thus blinded. All data were analyzed using unbiased statistical methods.

Reporting for specific materials, systems and methods

We require information from authors about some types of materials, experimental systems and methods used in many studies. Here, indicate whether each material, system or method listed is relevant to your study. If you are not sure if a list item applies to your research, read the appropriate section before selecting a response.

Materials & experimental systems

n/a	Involved in the study
☐	☒ Antibodies
☐	☒ Eukaryotic cell lines
☒	☐ Palaeontology and archaeology
☐	☒ Animals and other organisms
☒	☐ Clinical data
☒	☐ Dual use research of concern

Methods

n/a	Involved in the study
☒	☐ ChIP-seq
☒	☐ Flow cytometry
☒	☐ MRI-based neuroimaging

Antibodies

Antibodies used

anti-ANT1 (ab110322, Abcam)
anti-ATP5a (ab14748, Abcam)
anti-ATP5b (A21351, ThermoFisher Scientific)
anti-Beta Actin (60008-1-Ig, Proteintech Group)
anti-COX4 (459600, ThermoFisher Scientific)
anti-PPIF (18466-1-AP, Proteintech Group)
anti-cytochrome c (556433,BD Biosciences)
anti-DRP1 (611112, BD Biosciences)
anti-FIS1 (10956-1-AP, Proteintech Group)
anti-FLAG (F1804, Sigma Aldrich)
anti-GPD2 (17219-1-AP, Proteintech Group)
anti-MFF (17090-1-AP, Proteintech Group)
anti-MFN1 (13798-1-AP, Proteintech Group)
anti-MFN2 (12186-1-AP, Proteintech Group)
anti-MID49 (16413-1-AP, Proteintech Group)
anti-MID51 (20164-1-AP, Proteintech Group)
anti-MTCOI (459600, ThermoFisher Scientific)
anti-MTFP1 (14257-1-AP, Proteintech Group)
anti-NDUFA9 (ab14713, Abcam)
anti-OPA1 (612607, BD Biosciences)
anti-p-DRP1(ser616) (3455S, Cell Signaling)
anti-SDHA (459200, Invitrogen)
anti-TOMM40 (18409-1-AP, Proteintech Group)
anti-Total OXPHOS cocktail (ab110411, Abcam)
anti-UQCRC2 (ab14745, Abcam)
anti-VINCULIN (26520-1-AP, Proteintech Group)
anti-Rabbit HRP Conjugated (a120-101p, Bethyl Laboratories)
anti-Mouse HRP Conjugated (a90-116p, Bethyl Laboratories)

Validation

anti-ANT1 (ab110322, Abcam) validated for WB by the manufacturer: https://www.abcam.com/adenine-nucleotide-translocator-1--2ant-1--2-antibody-5f51bb5ag7-ab110322.html
anti-ATP5a (ab14748, Abcam) validated for WB by the manufacturer: https://www.abcam.com/adenine-nucleotide-translocator-1--2ant-1--2-antibody-5f51bb5ag7-ab110322.html
anti-ATP5b (A21351, ThermoFisher Scientific) validated for WB by the manufacturer: https://www.abcam.com/adenine-nucleotide-translocator-1--2ant-1--2-antibody-5f51bb5ag7-ab110322.html
anti-Beta Actin (60008-1-Ig, Proteintech Group) validated for WB by the manufacturer : https://www.ptglab.com/products/Pan-Actin-Antibody-66009-1-Ig.htm anti-COX4 (459600, ThermoFisher Scientific) validated for WB by the manufacturer: https://www.abcam.com/adenine-nucleotide-translocator-1--2ant-1--2-antibody-5f51bb5ag7-ab110322.html
anti-PPIF (18466-1-AP, Proteintech Group) validated for WB by the manufacturer and in our study by Crispr/Cas9 ablation in MEFs The band corresponding to PPIF was absent in PPIF knockout cells were generated (which themselves had been independently validated as being genetically disrupted by PCR and Sanger Sequencing).
anti-cytochrome c (556433,BD Biosciences) validated for WB by the manufacturer: https://www.bdbiosciences.com/ko-kr/products/reagents/western-blotting-and-molecular-reagents/western-blot-reagents/purified-mouse-anti-cytochrome-c.556433
anti-DRP1 (611112, BD Biosciences) validated for WB by the manufacturer and in our previous study (PMID: 34014035)
anti-FIS1 (10956-1-AP, Proteintech Group) validated for WB by the manufacturer and in our previous study (PMID: 34014035) anti-FLAG (F1804, Sigma Aldrich) validated for WB by the manufacturer and in our previous study (PMID: 34014035)
anti-GPD2 (17219-1-AP, Proteintech Group) validated for WB by the manufacturer: https://www.ptglab.com/products/GPD2-Antibody-17219-1-AP.htm
anti-MFF (17090-1-AP, Proteintech Group) validated for WB by the manufacturer: https://www.ptglab.com/products/MFF-Antibody-17090-1-AP.htm
anti-MFN1 (13798-1-AP, Proteintech Group) validated for WB by the manufacturer: https://www.ptglab.com/products/MFN1-Antibody-13798-1-AP.htm anti-MFN2 (12186-1-AP, Proteintech Group) validated for WB by the manufacturer: https://

www.ptglab.com/products/MFN2-Antibody-12186-1-AP.htm anti-MID49 (16413-1-AP, Proteintech Group) validated for WB by the manufacturer: https://www.ptglab.com/products/SMCR7-Antibody-16413-1-AP.htm anti-MID51 (20164-1-AP, Proteintech Group) validated for WB by the manufacturer: https://www.ptglab.com/products/SMCR7L-Antibody-20164-1-AP.htm anti-MTCOI (459600, ThermoFisher Scientific) validated for WB by the manufacturer: https://
www.thermofisher.com/antibody/product/COX4-Antibody-clone-1D6E1A8-Monoclonal/459600
anti-MTFP1 (14257-1-AP, Proteintech Group) validated for WB by the manufacturer and in our study in knockout mice tissues.
anti-NDUFA9 (ab14713, Abcam) validated for WB by the manufacturer: https://www.abcam.com/ndufa9-antibody-20c11b11b11-ab14713.html
anti-OPA1 (612607, BD Biosciences) validated for WB by the manufacturer and in our previous study (PMID: 34014035)
anti-p-DRP1(ser616) (3455S, Cell Signaling) validated for WB by the manufacturer and in our previous study (PMID: 34014035)
anti-SDHA (459200, Invitrogen) validated for WB by the manufacturer: https://www.thermofisher.com/antibody/product/SDHA-Antibody-clone-2E3GC12FB2AE2-Monoclonal/459200 anti-TOMM40 (18409-1-AP, Proteintech Group) validated for WB by the manufacturer: https://www.ptglab.com/products/TOMM40-Antibody-18409-1-AP.htm
anti-Total OXPHOS cocktail (ab110411, Abcam) validated for WB by the manufacturer and in our previous study (PMID: 34014035)
anti-UQCRC2 (ab14745) validated for WB by the manufacturer and in our previous study (PMID: 34014035)
anti-VINCULIN (26520-1-AP, Proteintech Group) validated for WB by the manufacturer: https://www.ptglab.com/products/Vinculin-Antibody-26520-1-AP.htm anti-Rabbit HRP Conjugated (a120-101p, Bethyl Laboratories) validated for WB by the manufacturer and in our previous study (PMID: 34014035)
anti-Mouse HRP Conjugated (a90-116p, Bethyl Laboratories) validated for WB by the manufacturer and in our previous study (PMID: 34014035)

Eukaryotic cell lines

Policy information about cell lines and Sex and Gender in Research

Cell line source(s)	Mouse embryonic fibroblasts (MFES) were generated in this study from wild type (Mtfp1+/+) and knockout (Mtfp1-/-) embryos, immortalized in culture with SV40 large T antigen and maintained in culture. U2OS osteosarcoma cells were obtained from commercial sources (ATCC) and HL-1 cardiomyocyte cells were obtained from the lab of Sigolene Meilhac (Institut Imagine/Universite Paris Cite) and were not independently authenticated. MTFPl-deficient U2OS cells were created by two rounds of Crispr/Cas9-mediated genome editing and were validated by Sanger sequencing and western blotting.
Authentication	Mouse embryonic fibroblasts were authenticated by PCR assay (see Supplementary Dataset 4 for primers). HL-1 and U2OS cells were not authenticated.
Mycoplasma contamination	Mycoplasma contamination verifications were routinely performed by PCR genotyping and sequencing (Eurofins).All cell lines tested negative for mycoplasma contamination.
Commonly misidentified lines (See ICLAC register)	No misidentified cell lines used in this study.

Animals and other research organisms

Policy information about studies involving animals; ARRIVE guidelines recommended for reporting animal research, and Sex and Gender in Research

Laboratory animals	Mus musculus musculus C57Bl6/NCrl were used in this study. The ages ranged from 8 to 34 weeks old and are specifically indicated in the figure legends.
Wild animals	This study did not use wild animals.
Reporting on sex	Both male and female mice were used in the in vivo studies demonstrating the progressive decline in cardiac function and death is non gender specific. Both genders were used for the ex vivo experiments: OMICS, ELISA, Histology, late stage bioenergetics (male mice); early stage bioenergetics, cell death assays, PTP assay (female mice).
Field-collected samples	This study did not involve samples collected from the field.
Ethics oversight	All animal experiments were performed according to French legislation in compliance with the European Communities Council Directives (2010/63/UE, French Law 2013-118, February 6, 2013) and those of Institut Pasteur Animal Care Committees (CETEA is Comité d'Ethique en Expérimentation Animale 89). The specific approved protocol numbers are 202005191046361 and 2018112017053431.

Note that full information on the approval of the study protocol must also be provided in the manuscript.
